# Acquisition of Flavescence Dorée Phytoplasma by *Scaphoideus titanus* Ball from Different Grapevine Varieties

**DOI:** 10.3390/ijms17091563

**Published:** 2016-09-15

**Authors:** Luciana Galetto, Dimitrios E. Miliordos, Mattia Pegoraro, Dario Sacco, Flavio Veratti, Cristina Marzachì, Domenico Bosco

**Affiliations:** 1 Istituto per la Protezione Sostenibile delle Piante, Consiglio Nazionale delle Ricerche (CNR), Strada delle Cacce 73, 10135 Turin, Italy; luciana.galetto@ipsp.cnr.it (L.G.); flavio.veratti@ipsp.cnr.it (F.V.); 2 Dipartimento di Scienze Agrarie, Forestali e Agroalimentari (DISAFA), Università degli Studi di Torino, Largo Paolo Braccini 2, 10095 Grugliasco, Italy; dim.miliordos@gmail.com (D.E.M.); mattia.pegoraro@unito.it (M.P.); dario.sacco@unito.it (D.S.); domenico.bosco@unito.it (D.B.)

**Keywords:** arneis, brachetto, dolcetto, freisa, moscato, timorasso, susceptibility

## Abstract

Flavescence dorée (FD) is a threat for wine production in the vineyard landscape of Piemonte, Langhe-Roero and Monferrato, Italy. Spread of the disease is dependent on complex interactions between insect, plant and phytoplasma. In the Piemonte region, wine production is based on local cultivars. The role of six local grapevine varieties as a source of inoculum for the vector *Scaphoideus titanus* was investigated. FD phytoplasma (FDP) load was compared among red and white varieties with different susceptibility to FD. Laboratory-reared healthy *S. titanus* nymphs were caged for acquisition on infected plants to measure phytoplasma acquisition efficiency following feeding on different cultivars. FDP load for Arneis was significantly lower than for other varieties. Acquisition efficiency depended on grapevine variety and on FDP load in the source plants, and there was a positive interaction for acquisition between variety and phytoplasma load. *S. titanus* acquired FDP with high efficiency from the most susceptible varieties, suggesting that disease diffusion correlates more with vector acquisition efficiency than with FDP load in source grapevines. In conclusion, although acquisition efficiency depends on grapevine variety and on FDP load in the plant, even varieties supporting low FDP multiplication can be highly susceptible and good sources for vector infection, while poorly susceptible varieties may host high phytoplasma loads.

## 1. Introduction

Flavescence dorée (FD), a severe grapevine yellows disease and a threat for wine production in many European viticulture areas [1], was first reported in Piemonte, northwestern Italy, in 1998 [2,3]. The disease is still present and epidemic in the southern part of the Region, between the Po valley and the Ligurian Appennin, which was recently (2014) included in the World Heritage Site list of Unesco (vineyard landscape of Piedmont: Langhe-Roero and Monferrato), on the ground of its “cultural landscape providing living testimony to winegrowing and winemaking traditions that stem from a long history” and its being an ‘outstanding example of man’s interaction with his natural environment’ [4]. Wine production in this area traditionally involves several cultivars (cv), and the possibility that very susceptible grapevine varieties may improve the efficiency of vector transmission, and therefore influence the disease epidemiology [5,6], is becoming a crucial question to address for management of FD in traditional grapevine growing areas of Piemonte. FD is caused by the FD phytoplasma (FDP), considered as a harmful organism in the EU and a quarantine pest.

Phytoplasmas are mollicutes that infect the phloem of many host plants and several organs of phloem-feeder insect vectors and actively multiply in both hosts [7]. Leafhoppers, planthoppers and psyllids transmit these pathogens in a persistent propagative manner, and phytoplasma diseases have been observed on hundreds of plant species all over the world [8]. In nature, FDP is transmitted by *Scaphoideus titanus* Ball [9], a monovoltine nearctic leafhopper, monophagous on grapevine. FDP is also routinely maintained under laboratory conditions in broad bean by *Euscelidius variegatus* Kirschbaum transmission [10]. This latter system is used to investigate details of FDP epidemiological cycle [11,12]. *Dictyophara europea* (Linneus), a polyphagous planthopper widespread in Europe [13], has also been reported to transmit FD from clematis to grapevine [14]. As this species preferentially feeds on amaranthus and nettle [15], its role as a vector in secondary spreading FD epidemics is unlikely, although it may represent a potential source of primary infection from outside the vineyard. In addition, the leafhopper *Orientus ishidae* (Matsumura) has been found positive for the presence of FDP [16,17,18], but, in the absence of successful transmission to healthy plants, it cannot be considered a vector of the disease.

The leafhopper vector *S. titanus* feeds on grapevines for its whole life and can acquire FDP as a nymph and as an adult. However, acquisition of FDP by nymph allows the vector to transmit the phytoplasma for a long period, whilst acquisition by adults, due to the long incubation period in the vector, results in a short period of infectivity, if any. The vector has only one generation per year and nymphs mainly develop from mid-May to mid-July, but it is well known that some eggs hatch later in the season, so nymphs can be recorded all through the month of August. On the contrary, adults can be found from the beginning of July until September [19,20]. Therefore, vector nymphs and adults may feed and eventually acquire FDP for a long period, during which phytoplasma titer varies significantly in the plant [21,22], thus potentially influencing the acquisition efficiency of the vector.

The first symptoms of FD infection are stunting and lack of bud break in May–June on most susceptible varieties [23]. During summer, infected grapevines show leaf yellowing or reddening, depending on the variety, downward leaf curling, drying of inflorescence and bunches, lack of cane lignification, presence of black spots on the new canes and premature leaf fall [24]. Symptoms may involve the entire plant or only selected branches. Besides the direct loss of grape production due to premature drying of the berries, the infected plant shows alterations in metabolism, energy processes, protein synthesis, protein fate, cellular and transport routes as well as cell defense and virulence [25], and the residual production, if any, has a poor quality. Infected plants may either die or recover, but they remain less productive for several years after the infection [26], although no information is available on the quality of wine produced from recovered vines. Grapevine is a plant species characterized by a very high intra-specific variability, largely exploited in viticulture to grow grapevines under very different climatic and soil conditions, with very different berry characteristics that allow the production of very diverse wines [27]. Although there are differences in the susceptibility of grapevine cultivars to FDP [26,28,29,30,31], most of the varieties used for wine production in Piemonte are highly susceptible ones, such as Arneis among the white cultivars and Barbera and Dolcetto among the red ones [22,23]. In particular, the local red cv Nebbiolo is more tolerant to FD, showing both milder symptoms and lower phytoplasma load compared to Barbera [22]. A recent study showed that FDP acquisition efficiency by *S. titanus* from Barbera and Nebbiolo grapevine varieties as well as the laboratory phytoplasma host (broad bean) is positively correlated to phytoplasma load in the source plant [6]. No grapevine varieties are known to be immune to the disease [32]. If the crop is the main source of infection, cvs that are poor hosts for vector acquisition should decrease the infection rate with important consequences on the disease epidemiology [33]. The knowledge of the suitability of the different cvs for vector acquisition may eventually suggest cv-specific control measures of FD. The objective of this work was to investigate the role of different grapevine varieties grown in the Piemonte Region as a source of inoculum for the vector *S. titanus*. This information is crucial to design a rational and effective control strategy for the containment of FDP epidemics.

## 2. Results

FDP load in infected source grapevines was measured by qPCR for 47 plants of the different varieties and expressed as the number of FDP cells per ng of plant DNA (Table 1).

**Figure 1:**
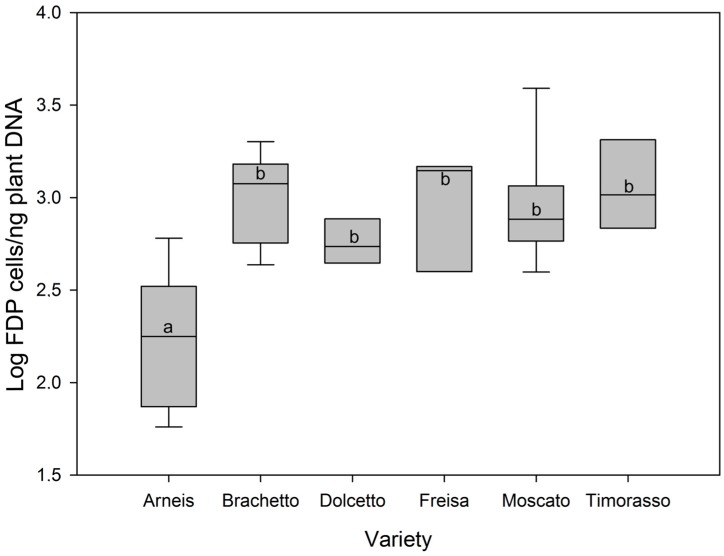
Mean Flavescence dorée phytoplasma loads in grapevines of different cultivars. Different letters refer to different mean values separated through the Holm–Sidak multiple comparison test.

A high variability of FDP load among plants of the same variety was observed, and the average phytoplasma population ranged from 677 to 1295 cells per ng of plant DNA in the different varieties except Arneis. The mean amount of FDP measured for Arneis (Figure 1) was significantly lower than those detected for all the other varieties (ANOVA, F = 10.752, total number of samples 47, with five degrees of freedom, *p* < 0.001, details of Holm–Sidak method, Table 1).

Following one week of feeding on FDP-infected grapevines and three-week latency on broad beans, *S. titanus* acquired FDP under field conditions from all the tested grapevine varieties, although with different efficiencies (Table 1). High proportions (34%–48%) of insects acquired FDP when caged on Arneis, Brachetto, Dolcetto and Freisa, while lower proportions were recorded for leafhoppers caged on FD-infected Timorasso and Moscato (22% and 9%, respectively). Leafhopper survival under the experimental conditions (from the beginning of the field acquisition until the end of the experiment four weeks later) varied from 40% to 70%, although the experimental setting was not designed to evaluate insect survival on the different grapevine varieties. Indeed, experimental vineyards were located in a restricted area of Piemonte, and they were all sloped, with an elevation ranging from about 200 to 400 m a.s.l., although with different aspects. We cannot exclude the possibility that minor differences in the vineyard location might have influenced leafhopper survival.

The generalized linear model showed a significant effect of the variety, a significant covariate effect of FDP load and a significant interaction (Table 2).

The parameter estimation of the GLM analysis is provided in Table 3.

Therefore, acquisition by the vector was significantly dependent on both the grapevine variety and the FDP load, as well as on their interaction. In particular, a significant interaction between variety and FDP load was observed for Arneis, Freisa and Timorasso varieties with respect to Dolcetto (Table 3).

## 3. Discussion

The experiments confirmed that *S. titanus* successfully acquired FDP from infected grapevines of six local varieties from the Piemonte Region, and demonstrated that efficiency depends on the grapevine variety, FDP load, and their interaction.

When considering the phytoplasma population in the different grapevine varieties, FDP mean loads varied from a few hundred to a few thousand cells per ng of grapevine DNA, according to the plant and the different varieties. However, since phytoplasma loads for the different varieties was estimated at different locations, the influence of different environmental conditions on FDP multiplication cannot be excluded. Different varieties are cultivated at different locations according to the product specifications for Denominazione di Origine Controllata and Denominazione di Origine Controllata e Garantita (DOC and DOCG) wines, and this hampered the possibility of testing the different cvs at the same location under productive field conditions.

Three red and three white varieties were used in FDP acquisition experiments. Among them, some are highly susceptible to FDP, e.g., Dolcetto and Arneis, while others, such as Timorasso and Moscato, are more tolerant. Statistical analysis of experimental data demonstrated that acquisition efficiency was dependent on the grapevine variety and on FDP load in the source plants. Moreover, there was a positive interaction for acquisition between variety and phytoplasma load and this interaction was significant for most of the varieties. A similar work was done on the two most important cultivars of the Piemonte Region, Barbera and Nebbiolo, and a clear relationship between phytoplasma load and vector acquisition efficiency was found [6].

A different feeding behavior/preference of the vector on/for different grapevine varieties might explain different acquisition efficiencies among the cultivars. However, no data are available to support this hypothesis for the six local cultivars from Piemonte, and a detailed analysis of *S. titanus* feeding behavior is so far available only for Cabernet Sauvignon [34]. Taken together, the data on FDP acquisition by *S. titanus* suggest that the vector can acquire FDP with high efficiency and thus spread the disease very fast in most of the varieties. In some grapevine cvs, e.g., Moscato and Timorasso, phytoplasma acquisition appears relatively inefficient, but more data, obtained under similar environmental conditions, are needed to confirm this finding.

In the Piemonte Region, high incidence of the disease is observed in areas where Barbera, Dolcetto, and Arneis are the prevalent varieties. These are among the most suitable cvs for vector acquisition (with acquisition efficiencies of 36%–59%; this work and [6]). On the other hand, infected plants are less frequent in Moscato, Timorasso and Nebbiolo cvs, which are the less suitable ones for vector acquisition (acquisition efficiencies of 9%–22%; this work and [6]). These results suggest that disease spread correlates more with vector acquisition efficiency than with the FDP load in the source grapevine. Indeed, infected plants of some varieties, like Timorasso and Moscato, host a relatively high number of phytoplasma cells, but very few plants are infected under field conditions. Accordingly, acquisition by the vector of these varieties was poor. On the contrary, plants of cv Arneis host low phytoplasma populations, and the disease incidence is generally high, consistent with high acquisition efficiency by the vector.

However, correlation between acquisition efficiency and disease spread can only explain secondary (vine to vine) spread of the disease within the vineyard. It is known that, in these last years in Piemonte, FD spread is also due to primary infections, sustained by incoming *S. titanus* adults that fed on source plants outside the vineyards, e.g., gone-wild rootstock vines infected by FDP (*Vitis riparia*, *V. berlandieri*, *V. rupestris* and their hybrids). Actually, many *S. titanus* occur in uncultivated areas surrounding vineyards, and several of these are infected by FDP [35]. Preliminary results from our laboratory confirm the role of infected leafhoppers from the wild compartment in the spread of the disease in Piemonte [36].

The grapevine genotype is likely to influence several aspects of FD epidemiology, among these are: (i) vector acquisition efficiency; (ii) phytoplasma multiplication; and (iii) symptom expression. It is well-known that there are no resistant grapevine genotypes, although some cultivars are less susceptible to the disease [22,32]. According to the cited literature, low susceptibility is associated with a low number of phytoplasma cells in the plant; however, this work provides evidence that even highly susceptible varieties, like Arneis, may support low FDP multiplication. Nevertheless, they are still good sources of infection for the vector. Therefore, even when cvs support low phytoplasma loads, their attitude as FD source plants must be evaluated and taken into consideration for the epidemiology of the disease. This also supports the phytosanitary measure of uprooting the infected plant at the first appearance of symptomatic vegetation On the other hand, poorly susceptible cvs like Timorasso and Moscato can host a relatively high phytoplasma load. These apparent contradictions highlight the complexity of the FDP–grapevine–*S. titanus* interactions that are regulated by a number of factors regarding the pathogen, the vector and the host plant. FD is caused by phytoplasmas of distinct 16S ribosomal subgroups (V-C and V-D, [37,38]), but no differences have been found so far in their behavior during infection of broad bean [12,39], the laboratory host of FD [10], or in their relationships with the vector [40]. However, a differential behavior of distinct FD genotypes during grapevine colonization has not been addressed. On the other hand, all European populations of *S. titanus* are genetically very homogeneous, consistent with a single or a few introductions of this insect from North America [41,42,43] and should have similar relationships with the phytoplasma. As for grapevine genetics, transcriptomic analyses of FDP-infected plants revealed differences in different varieties [25,44,45], a reflection of the diverse response strategies to microbial infections of different grapevine varieties [46]. Moreover, recent data indicate that, among environmental parameters, at least temperature clearly influences FDP multiplication in plants and vectors [12,39]. Plant genetics and environmental conditions are probably the most important factors determining the success of host colonization by FDP.

## 4. Materials and Methods

### 4.1. Vector and Flavescence Dorée Phytoplasma Source Plants

For all the experiments, *S. titanus*, a vector of FDP under natural conditions, was used. Vector colonies were established according to [24]. Two-year-old branches bearing leafhopper eggs were collected in vineyards during winter, cut into 20–30 cm long pieces and kept in plastic bags in a cold room at 4 °C. To allow egg hatching, 8–10 kg of wood pieces were put inside cubical, insect-proof, screen houses (100 cm × 100 cm × 100 cm) in a glasshouse with natural light and temperature ranging from 20 to 30 °C. The wood pieces were placed over a thin layer of a plastic carpet, and were periodically humidified in order to avoid egg dehydration. Potted grapevine cuttings together with potted broad beans (*Vicia faba* L.) were introduced in the screen house and replaced every 3 weeks. Egg hatching started about 30–40 days after the introduction of the branches in the cage. Insect nymphs were periodically collected and used for the experiments.

**Figure 2:**
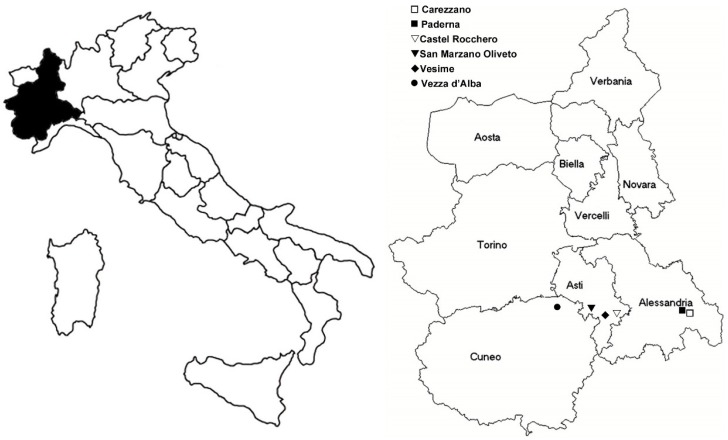
Piemonte Region: localization of the different vineyards under analysis for field acquisition experiments of Flavescence dorée phytoplasma by *Scaphoideus titanus*.

Vineyards of different viticultural areas of Piemonte Region, Italy, were monitored for the presence of FD and the following were selected for the FDP acquisition experiments: Carezzano (cv Timorasso; 44.817081, 8.904196, slightly sloped, row orientation: North-West–South-East (NW–SE), elevation: 300 m above sea level (a.s.l.), aspect: East (E)) and Paderna (cv Freisa; 44.826099, 8.894926, slightly sloped, row orientation: North–South (N–S), elevation: 300 m a.s.l.; aspect: North (N)) in Alessandria province, Castel Rocchero (cv Dolcetto; 44.729536, 8.421548, sloped, row orientation: N–S, elevation: 400 m a.s.l., aspect: West (W)), San Marzano Oliveto (cv Moscato; 44°45′26.4″ N 8°18′47.0″ E, sloped, row orientation: NW–SE, elevation: 300 m a.s.l., aspect: North–East (NE)) and Vesime (cv Brachetto; 44.619363, 8.211306, sloped, row orientation: NE–South West (SW), elevation: 225 m a.s.l., aspect: SW) in Asti province and Vezza d’Alba (cv Arneis; 44.777526, 7.971033, sloped, row orientation: NW–SE, elevation: 400 m a.s.l., aspect: NE) in Cuneo province (Figure 2).

Arneis, Moscato and Timorasso are white varieties, and the others are red ones. Basal, median and apical leaves of symptomatic grapevine shoots were sampled according to [22], and analyzed for the presence of FDP by nested PCR and PCR followed by restriction fragment length polymorphism analyses, as detailed below. Following FDP diagnosis, infected plants were labelled for successive acquisition experiments.

### 4.2. Flavescence Dorée Phytoplasma Acquisition Experiments

Acquisition experiments were performed during the vegetative seasons of 2013–2015. All the acquisition experiments were carried out between mid-July and mid-August as, in this period, FDP load in the plant is higher [22] and acquisition by the vector is more efficient [6].

For acquisition from FD-infected grapevines of the different varieties, vector nymphs were fed on four plants of Timorasso, three of Arneis, three of Freisa, two of Brachetto, two of Dolcetto and five of Moscato. All plants were previously identified as infected based on symptom observation and PCR analyses (as described below). About 50 third-fifth instar nymphs of *S. titanus* were caged inside a net cage on a symptomatic grapevine branch of an infected plant. After seven day-acquisition access period (AAP) under field conditions, caged branches were cut from the plant and brought to the laboratory where the insects were collected and transferred to broad bean plants, as these are suitable herbaceous hosts for long-term maintenance of *S. titanus* and are routinely used for both FD acquisition and completion of latency period (LP, [47]). LP lasted for 3 weeks and was carried out in a climatic chamber at 24 °C, light:dark 16:8 h. All insects collected from the same branch were caged together on the same broad bean plant and, at the end of the latency, all insects were singularly tested by PCR for the presence of FDP, as described later.

### 4.3. DNA Extraction and Phytoplasma Detection by PCR

Total DNA was extracted from grapevine leaf midribs (1.5 g), and single insects according to [48]. For FDP diagnosis, 2 µL of DNA was used in direct PCR with universal primers P1/P7 [49]. Reaction products were used as templates in nested PCRs driven by primers R16(V)F1/R1 [50]. Reaction and cycling conditions were as detailed in the original papers. PCR products were separated in a 1% agarose gel, buffered in TBE (90 mM Tris borate and 2 mM EDTA, pH 8.3), stained with ethidium bromide and visualized under UV light.

### 4.4. Flavescence Dorée Phytoplasma Quantification by qPCR

FDP load in infected source grapevines was measured as number of FDP cells per ng of plant DNA, as previously detailed [22,51]. To quantify FDP and grapevine DNAs, FdSecyFw/Rv and Vitis18SF1/R1 [22] were used, respectively. Standard curves for the absolute quantification of FDP and host DNA were obtained by dilution of (i) plasmid p26SecYFD, containing the appropriate secY gene target sequence from a local isolate of FDP; and (ii) total DNA extracted from healthy plants, as described in [22]. Plant sample DNAs were diluted in ddH_2_0 to a final concentration of 1 ng/µL and used (5 µL) as template in real-time assays together with iQTM SYBR Green Supermix (Bio-Rad Laboratories, Hercules, CA, USA) and specific primer pairs at a final concentration of 300 nM, in a volume of 25 µL. The PCR was performed in 96-well plates in a CFX Connect Real-Time PCR (Bio-Rad Laboratories, Hercules, CA, USA) thermal cycler, following cycling conditions described by [22]. Each sample was run in triplicate in the same plate. For each PCR, water instead of DNA was used as negative control. Threshold levels, threshold cycles, and standard curves were automatically calculated by the Bio-Rad CFX Manager software, version 3.0. Specificity of the reaction was tested by running melting curves of the amplicons, following each quantification reaction.

FDP was quantified in 4 plants of cv Timorasso, 12 plants of cv Arneis, 3 plants of cv Freisa, nine plants of cv Brachetto, 7 plants of cv Dolcetto and 12 plants of cv Moscato, including those used for acquisition experiments (source plants). Plants of different cultivars were all from the same vineyards, as detailed above.

### 4.5. Data Analyses

FDP loads measured in source grapevines of all cultivars were analyzed. Since raw data of phytoplasma loads were not normally distributed and variances were not homogeneous, they were log-transformed before analysis. To compare the phytoplasma loads measured in all plants of different varieties, one-way ANOVA was performed. When significant, mean values were separated through a Holm–Sidak multiple comparison test.

The effects of plant variety and FDP titer on the FDP acquisition efficiency by the vector (expressed as the number of PCR-positive insects following feeding on different cultivars) were analyzed with a generalized linear model based on a binomial distribution and a logit function as link. The variables were grapevine cultivar as a factor, FDP load as covariate, and their interaction. All statistical tests were performed using SPSS ver. 20.0.0 (2011) (IBM Corp. Armonk, New York, NY, USA).

## Figures and Tables

**Table 1:** Mean Flavescence dorée phytoplasma (FDP) load measured for different cultivars, expressed as FDP cells/ng plant DNA ± Standard Error, and acquisition from different grapevine varieties expressed as FDP-PCR-positive *Scaphoideus titanus* out of total tested insects.

Variety	Number of Plants Used for FDP Quantification	FDP Load *	Number of Plants Used for Acquisition	PCR Positive/Total Tested Insects
Arneis	12	230.50 ± 55.88 ^a^	3	45/132
Brachetto	9	1136.19 ± 177.37 ^b^	2	16/41
Dolcetto	7	677.17 ± 171.54 ^b^	2	30/62
Freisa	3	1090.00 ± 346.58 ^b^	3	37/89
Moscato	12	1216.81 ± 386.59 ^b^	5	16/178
Timorasso	4	1294.70 ± 378.72 ^b^	4	22/101

* Different letters refer to different mean values separated through the Holm–Sidak multiple comparison test.

**Table 2:** Output of the generalized linear model (GLM) analysis. df: degree of freedom, Sign.: significance, FDP: Flavescence dorée phytoplasma.

Source	Type III		
	Wald Chi-Square	df	Sign.
(Intercept)	27.892	1	0.000
Variety	26.796	5	0.000
FDP	13.788	1	0.000
Variety× FDP	19.628	5	0.001

**Table 3:** Parameter estimation of the Generalized Linear Model Analysis. Model: (intercept), Variety, FDP, Variety × FDP. FDP: Flavescence dorée phytoplasma loads measured as phytoplasma cells/ng plant DNA; Events: acquisition efficiency.

Parameter	Parameter Estimation	95% Wald Confidence Interval		Significance
		Upper	Lower	
Intercept	0.432	−0.544	1.408	0.385
Arneis	−3.590	−5.377	−1.802	0.000
Brachetto	−2.801	−6.339	0.738	0.121
Freisa	−2.023	−3.565	−0.482	0.010
Moscato	−2.457	−3.992	−0.923	0.002
Timorasso	−3.538	−5.148	−1.929	0.000
Dolcetto	0 ^a^	–	–	–
FDP	−0.001	−0.003	0.001	0.248
ARNEIS × FDP	0.023	0.010	0.035	0.000
BRACHETTO × FDP	0.003	−0.001	0.007	0.109
FREISA × FDP	0.002	6.342 × 10^−5^	0.005	0.044
MOSCATO × FDP	0.001	−0.002	0.003	0.554
TIMORASSO × FDP	0.002	0.000	0.005	0.024
DOLCETTO × FDP	0 ^a^	–	–	–

^a^ Considered as reference and included in the intercept estimation.
